# Corneal endothelial cell density and associated factors among adults at a regional referral hospital in Uganda: a cross-sectional study

**DOI:** 10.1186/s12886-024-03435-4

**Published:** 2024-04-15

**Authors:** Shamiim Namwase, Sam Ruvuma, John Onyango, Teddy Kwaga, Abel Ebong, Daniel Atwine, David Mukunya, Simon Arunga

**Affiliations:** 1 Department of Ophthalmology, Mbarara University of Science and Technology, Mbarara, Uganda; 2 Soar Research Foundation, Mbarara, Uganda; 3 London School of Hygiene & Tropical Medicine, London, UK; 4 Dr. Arunga’s Eye Hospital, Mbarara, Uganda; 5 Department of Community and Public Health, Busitema University, Mbale, Uganda; 6 Department of Research, Nikao Medical Center, Kampala, Uganda

**Keywords:** Cornea, Endothelial cell density, Uganda

## Abstract

**Background:**

To assess the prevalence of low corneal endothelial cell density and correlates of corneal endothelial cell density among adults attending Mbarara University and Referral Hospital Eye Centre in Uganda.

**Methods:**

In this hospital-based cross-sectional study, participants 18 years and older, were enrolled. We obtained informed consent, and basic demographic data. We also conducted visual acuity, a detailed slit lamp examination, intra-ocular pressure, corneal diameter, tear-film break-up time, keratometry, A-scan, and pachymetry on all participants. A confocal microscope Heidelberg HRT3 was used to examine the central cornea and to obtain the mean cell density (cells/mm^2^). To calculate the proportion of low endothelial cell density, descriptive statistics were used, whereas correlates of endothelial cell density were assessed, using linear regression analyses.

**Results:**

We evaluated a total of 798 eyes of 404 participants aged between 18 and 90 years (males = 187, females = 217). The average endothelial cell density was 2763.6 cells/mm^2^, and there was a decrease in endothelial cell density with increasing age, irrespective of gender. There was no significant difference in endothelial cell density between males and females. Increasing age (adjusted coefficient − 10.1, *p* < 0.001), history of smoking (adjusted coefficient − 439.6, *p* = 0.004), history of ocular surgery (adjusted coefficient − 168.0, *p* = 0.023), having dry eye (adjusted coefficient − 136.0, *p* = 0.051), and having arcus senilis (adjusted coefficient − 132.0, *p* = 0.08), were correlated with lower endothelial cell density. However, increasing corneal diameter (adjusted coefficient 134.0, *p* = 0.006), increasing central corneal thickness (adjusted coefficient 1.2, *p* = 0.058), and increasing axial length (adjusted coefficient 65.8, *p* = 0.026), were correlated with higher endothelial cell density. We found five eyes (0.63%) from different participants with a low endothelial cell density (< 1000cells/mm^2^).

**Conclusion:**

Our study established baseline normal ranges of ECD in a predominantly black African population, and found that low ECD is rare in our population. The elderly, smokers, and those with past ocular surgery are the most vulnerable. The low prevalence could be due to a lack of reference values for the black African population.

## Background

The cornea, a transparent avascular structure in the eye, contributes significantly to the eye’s optical power and serves a protective role by shielding intraocular structures from the external environment [1]. Within the cornea, the endothelium plays a critical role in maintaining the eye’s optical transparency and hydration [1]. Despite the endothelium’s importance, there is a lack of data on the prevalence of low corneal endothelial cell density, both globally, and in Uganda. Reduced endothelial cell density can lead to corneal decompensation, causing issues like corneal edema, bullous keratopathy, reduced visual acuity, and severe pain [2]. Additionally, long-standing corneal edema can increase the risk of complications, such as; vascularization, infection, and scarring [2]. Understanding the prevalence and factors associated with low endothelial cell density is vital for clinical assessment and patient care. Therefore, this study aimed at assessing the prevalence of low endothelial cell density, and its correlates in a predominantly African population.

## Methods

### Study design

We conducted a hospital-based cross-sectional study, to assess the prevalence of low corneal endothelial cell density and correlates of corneal endothelial cell density among adults attending Mbarara University and Referral Hospital, Eye Centre (MURHEC), a tertiary level public eye hospital in Mbarara, Southwestern Uganda.

### Case definition

Low corneal endothelial cell density was defined as endothelial cell density (ECD) of less than 1000 cells/mm^2^, in accordance to a study by Yamaguchi et al. [3].

### Inclusion and exclusion criteria

#### Inclusion criteria

All adults 18 years and above, for whom confocal microscopy was deemed suitable.

#### Exclusion criteria

A patient was considered ineligible to participate in the study if they had; corneal perforation, dense corneal scars, painful eye conditions, or poor corneal visibility. These criteria were based on existing literature [1, 4].

#### Data collection

On every clinic day during the study period from January 2022 to March 2022, clinical staff identified eligible patients and called upon the research assistant to recruit them.

Written informed consent was obtained from study participants in their preferred language. We then used a structured interviewer-administered questionnaire to collect demographics and medical history. We measured presenting and pinhole visual acuity using Snellen`s acuity chart for patients who could read and used the tumbling E-chart for participants unable to read. Fluorescein staining was done, to identify any epithelial defects, and to confirm clinical diagnosis of dry eye. This was carried out before instilling other drops. A detailed slit lamp examination of the ocular structures was done, and observations were systematically recorded. A dilated fundoscopy, using a 90 diopter condensing lens was performed.

We measured corneal diameter, using a slit lamp (the geometrical mean of the horizontal and vertical diameters was taken). Tonometry was done for patients with suspicious cup disc ratio, defined as cup disc ratio of 0.5 or more, or asymmetry of > 0.2, using Goldman’s applanation tonometer, after instilling 1–2 drops of amethocaine 0.5%. Pachymetry using a DGH Ultrasonic Tachymeter (Pachette 2 DGH-550) was done, to measure central corneal thickness. K readings and anterior chamber depth were measured, using an auto refractor, and A-Scan Ophthalmic Ultrasound, respectively.

A confocal microscope (HRT3 with Rostock Corneal Module [RCM], Heidelberg Engineering, Heidelberg, Germany) was used to examine the central corneal endothelium, after application of amethocaine eye drops, and obtained the mean cell density (cells/mm^2^). Images from the central cornea were captured, and the best image was then selected. The centers of 50 contiguous cells were marked manually, and were analyzed by a built-in software program. The mean cell density (MCD) was automatically calculated, and displayed. Data were collected from both the right and left eye of the participants. 5 patients had measurements from only one eye because the second eye had atleast one of the following;

- Two patients had corneal scars.
- Two patients had painful eye conditions (uveitis).
- One patient declined measurement from the 2nd eye due to discomfort.

The appropriate management and treatment according to MURHEC management protocols was then given.

### Data analysis

All questionnaires were checked for completeness, prior to entry into the database designed using EPI-Info software version 7.2. We implemented appropriate data cleaning and data verification processes before analysis. This was done by cross-checking the participants’ medical records.

Data were exported to Stata software version 15.0, College Station, Texas, USA for analysis. Participants’ categorical data was then summarised, as frequencies and percentages. Continuous data were summarised as means/medians with standard deviation/interquartile range.

The prevalence of low corneal endothelial cell density (ECD) was calculated as a fraction of all eyes with low corneal ECD, out of all those enrolled in the study, and expressed as a percentage. Five participants had measurements from only one eye and these were dropped from the analysis. In our study, low corneal ECD was taken as any count < 1000 cells/mm^2^ [3].

To establish the average corneal ECD, the geometric mean of the ECD measurements from both the right and left eye was calculated for each participant, as a square root of the product of the readings from both eyes. The geometric mean was used as a single measure of ECD that represented each participant. Summary statistics were generated for the geometric means of the corneal endothelial cell density, that is mean and standard deviation. Further stratification was performed across categories of age and gender, and results were presented using a line graph.

To determine the correlates of corneal endothelial cell density, we used ECD as a continuous dependent variable in this analysis. All participants’ characteristics were used as independent variables. The normality of the dependent variable was examined. In bivariable analyses, simple linear regression was performed, to establish the correlation between each independent variable with ECD. Unadjusted beta-coefficients with their corresponding 95% confidence intervals were reported. A value was considered significant if *p* < 0.05.

All variables that showed statistically significant correlation with ECD in bivariable analyses (*p* < 0.1), and those with biological correlation were considered for multivariable analysis. Assumptions of multiple linear regression were checked. These included absence of multicollinearity among independent variables, normality of residuals, and heterogeneity among the independent variables (Heteroskedasticity). Multiple linear regression was performed, so as to control confounding among the correlates of ECD. Adjusted beta-coefficients with their corresponding 95% CI and *p* values were reported. A value was considered significant, if *p* < 0.05.

## Results

### Participant characteristics

During the period from January 2022 to March 2022, 525 participants were screened for eligibility from Mbarara University and Referral Hospital, Eye Centre. Of these, 404 were enrolled into the study, while 121 were excluded. Among the excluded, 31 had poor corneal visibility, 43 corneal ulcers, 26 had corneal scars, and 21 painful eye conditions.

Table 1 shows socio-demographic, medical, surgical and behavioral characteristics of the participants. The overall mean age of the participants was 47 years (SD 18.5). Most of the participants were females 217 (53.7%). Majority of participants worked outdoor 290 (72.0%).

**Table 1:** Baseline characteristics of participants, *N* = 404

Characteristic	n (%)
Sex	
Male	187 (46.3)
Female	217 (53.7)
**Age categories**	
18–30	101(25.0)
31–40	72 (17.8)
41–50	54 (13.4)
51–60	70 (17.3)
61–70	57 (14.1)
> 70	50 (12.4)
**Occupation** ^**b**^	
Outdoor	290 (72.0)
Indoor	113 (28.0)
History of eye drop use prior to presentation	181(45.0)
History of ocular trauma	54 (13.4)
History of ocular surgery^c^	51(12.6)
History of diabetes	39 (9.7)
History of smoking	10 (2.5)
History of radiotherapy	01(0.3)
History of contact lens wear	01(0.3)

^b^n = 403 due to missing data. The participants who spent most of their day working outside were considered as “outdoor” and those who spent most of their day working indoors were considered as “indoor”. ^c^History of ocular surgery included cataract surgery (36[8.9%]), glaucoma surgery (8[2.0%]), conjunctival excisions (7[1.7%]) and scleral buckling (1[0.3%])

Table 2 shows clinical presentation of the participants. Majority of participants had vision in the better eye of 6/12 (271 [67.1%]). The most common examination finding was conjunctival hyperemia RE 100 (24.8), LE 92 (22.8). The mean IOP was RE 15.0 (6.0), LE 14.8 (5.8), mean corneal diameter RE 10.9 (0.6), LE 10.9 (0.6), keratometry RE 43.9 (SD = 2.4), LE 43.7 (SD = 2.1), mean CCT RE 522.9 (SD = 39.0), LE 524.8 (37.7), mean axial length RE 22.8 (SD = 1.1), LE 22.7 (0.9), and mean ACD RE 2.9 (SD = 0.4), LE 2.9 (SD = 0.4).

**Table 2:** Clinical presentation, findings and investigations, *N* = 808

Variable	n (%)	n (%)
**Presenting VA in better eye**		
6/5 to 6/12	271(67.1)	
> 6/12 to 6/18	49 (12.1)	
> 6/18 to 6/60	59 (14.6)	
> 6/60 to 3/60	14 (3.5)	
> 3/60	11 (2.7)	
**Presenting VA in worse eye**		
6/5 to 6/12	203 (50.2)	
> 6/12 to 6/18	63 (15.6)	
> 6/18 to 6/60	56 (13.9)	
> 6/60 to 3/60	45(11.1)	
> 3/60	37 (9.2)	
**Examination findings**	**RE**	**LE**
Conjunctival hyperemia	100 (24.8)	92 (22.8)
Pterygium	51 (12.7)	45 (11.2)
Arcus senilis	189 (39.5)	189 (39.5)
Cells in A/C	12 (3.0)	13 (3.2)
Pseudoexfoliation	89 (22.1)	80 (19.8)
Lens opacification	188 (46.5)	175 (43.3)
Vitreous degeneration	19 (4.9)	15 (3.9)
Retinal hemorrhage	07 (1.7)	07 (1.7)
Retinal detachment	06 (1.5)	05 (1.2)
Retinal exudates	01 (0.3)	02 (0.5)
Mean IOP(SD)*	15.0 (6.0)	14.8 (5.8)
Mean Corneal diameter (SD)*	10.9 (0.6)	10.9 (0.6)
Tear film instability	48 (12.1)	44 (11.1)
**Diagnostic parameters**		
Mean CCT (SD)*	522.9 (39.0)	524.8 (37.7)
Mean Keratometry (SD)*	43.9 (2.4)	43.7 (2.1)
Mean AXL (SD)*	22.8 (1.1)	22.7 (0.9)
Mean ACD (SD)*	2.9 (0.4)	2.9 (0.4)

Where * is indicated, *n* < 808 due to missing data. For IOP (*n* = 796), Corneal diameter (*n* = 782), CCT (*n* = 806), Keratometry (*n* = 792), AXL (*n* = 800), ACD (*n* = 796). Mean keratometry and mean corneal diameter were calculated as geometrical means from K1 and K2 readings and from horizontal and vertical corneal diameters respectively.

Presenting visual acuity was graded according to WHO grading of visual impairment. There was a significant difference in the analysis per eye that compared the mean ECD between VA in the better and worse eye in both the RE and LE. Overall, the eyes with worse VA had a low mean ECD (RE = 2584.2 ± 517.3 cells/mm^2^, LE = 2531.4 ± 539.2 cells/mm^2^) as compared to those who had better VA (RE = 2926.6 ± 535.5 cells/mm^2^, LE = 2951.9 ± 504.2 cells/mm^2^), p < 0.001

### Prevalence of low corneal endothelial cell density

**Fig. 1:**
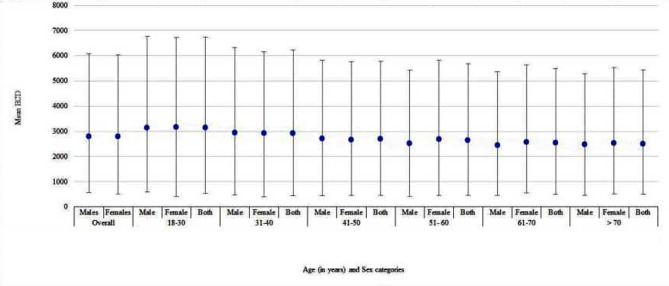
A graph of moving averages for the geometric mean ECD at each age category by gender The dots represent mean of the geometric mean endothelial cell densities at a specific age group, and with respect to gender, and the whiskers represent standard deviations

The prevalence of low corneal ECD (endothelial cell density < 1000cells/mm^2^) was found to be 5 out of 798 eyes (0.63%). These 5 eyes were from different participants. The geometric mean ECD among all 399 participants with measurements in both eyes was 2763.6 ± 535.3cells/mm^2^. The geometric mean ECD was 2761.3 ± 565.8cells/mm^2^ and 2765.6 ± 509.0 cells/mm^2^ in male and female, respectively (Fig. 1). Using a ttest to compare equality of the mean ECD across the 2 eyes, the mean ECD in the right eye was 2773.7 ± 554.2cells/mm^2^, and in the left eye was 2766.2 ± 559.2cells/mm^2^ (*p* = 0.589).

### Correlates of corneal endothelial cell density

To determine the correlates of corneal endothelial cell density, a simple linear regression model presented in Table 3, was applied. The variables that were strongly associated with reduction in ECD included Age (adjusted Coefficient − 10.1, 95% CI [-13.1 to -7.1], *P* < 0.001), history of smoking (adjusted Coefficient − 439.6, 95% CI [-741.7 to -137.6, *P* = 0.004), history of ocular surgery (adjusted Coefficient − 168.0, 95% CI [-312.5 to -23.5], *P* = 0.023), tear film instability (adjusted Coefficient − 136.7, 95% CI [-286.5 to -17.3], *P* = 0.051), and arcus senilis (adjusted Coefficient − 132.0, 95% CI [-283.6 to 19.5], *P* = 0.088). On the other hand, the variables which were strongly associated with a higher ECD included corneal diameter (adjusted Coefficient 134, 95% CI [39.0 to 229], *P* = 0.006), central corneal thickness (adjusted Coefficient 1.2, 95% CI [-0.04 to 2.5], *P* = 0.058), and axial length (adjusted Coefficient 65.8, 95% CI [7.9 to 123.8], *P* = 0.026).

### Linear regression analysis of correlates of ECD

**Table 3:** Results of bi-variable and multivariable analysis of correlates of ECD

Variable	Bi-variable Analysis			Multivariable Analysis		
	Unadjusted Coefficient	[95% Confidence Interval]	*P* value	Adjusted Coefficient	[95% Confidence Interval]	*P* value
**Sex**						
Female	4.3	-101.5, 110.1	0.9365	70.3	-27.1, 167.6	0.157
Male	Ref					
Age	-12.9	-15.4, -10.3	< 0.001	-10.1	-13.1, -7.1	< 0.001
Occupation						
Indoor	183.6	67.4, 299.7	0.0020			
Outdoor	Ref					
History of ocular trauma						
Yes	167.1	13.7, 320.4	0.0328			
No	Ref					
History of smoking						
Yes	-523.7	-857.2, -190.2	0.0022	-439.6	-741.7, -137.6	0.004
No	Ref					
History of diabetes						
Yes	-189.0	-367.8, -10.3	0.0382			
No	Ref					
History of ocular surgery						
Yes	-360.6	-618.5, -260.6	< 0.001	-168.0	-312.5, -23.5	0.023
No	Ref					
History of eye drop use						
Yes	35.9	-54.1, 246.8	0.5067			
No	Ref					
Pterygium						
Yes	39.1	-103.7, 181.9	0.5905			
No	Ref					
Arcus Senilis						
Yes	-234.9	-401.7, -68.1	0.0059	-132.0	-283.6, 19.5	0.088
No	Ref					
Pseudoexfoliation						
Yes	-430.4	-783.1, -77.7	0.0169			
No	Ref					
Lens opacification						
Yes	-376.8	-492.7, -260.9	< 0.001			
No	Ref					
Vitreous degeneration						
Yes	-392.7	-651.0, -134.4	0.0030			
No	Ref					
Tear film Instability						
Yes	-250.4	-406.5, -94.4	0.0017	-136.7	-286.5, -17.3	0.051
No	Ref					
IOP	8.7	-2.5, 20.0	0.1274			
Corneal diameter	204.4	102.2, 306.5	0.0001	134.0	39.0, 229.0	0.006
CCT	2.5	1.1, 4.0	0.0008	1.2	-0.04, 2.5	0.058
Keratometry	-28.1	-50.5, -5.6	0.0145			
AXL	65.2	5.7, 124.7	0.0318	65.8	7.9, 123.8	0.026
ACD	152.9	11.1, 294.7	0.0346			

## Discussion

Our study aimed to determine the prevalence of low corneal endothelial cell density and correlates of corneal ECD in a predominantly African population. We found a prevalence of low ECD of 5/798 (0.63%) in our population. Although we didn’t find any available literature for comparison, a prevalence of 0.63% shows that low ECD is rare in our region.

The mean ECD of 2763.6 ± 535.3cells/mm^2^ in our study was comparable to the average ECD, reported in other studies of somewhat similar populations. In Pakistan, Malaysia, Nigeria and Egypt, they found the average ECD as follows: 2654 ± 341/mm^2^, 2648 ± 310 cell/mm², 2,610.26 ± 371.87 cells/ mm² and 2647.50 ± 382.62 cells/mm^2^_,_ respectively.

We also explored correlates of ECD. We found that age, tear film instability, history of previous ocular surgery, history of smoking, central corneal thickness, corneal diameter, arcus senilis, and axial length were the most important factors that influence ECD.

The results revealed a decrease in ECD with increasing age. For example, the average ECD was 3113.1cells/mm^2^ in the 18–30 years group, decreasing to 2472 cells/mm^2^ in the above 70 years age group. It is important to note that age comes along with related diseases such as cataract and glaucoma, which will eventually require surgery, and age is also a risk factor for low ECD. Therefore, people in this age group should be considered a high-risk group, and clinicians should be mindful of this during pre-operative assessment, and counsel older persons appropriately.

Our study found that persons with tear film instability (dry eye) were more likely to have a lower corneal ECD. This is due to ocular surface inflammation, seen in dry eye disease, that leads to reduced corneal innervation, which eventually leads to reduced corneal ECD [5].

History of previous surgery increased the chances of a patient having a low ECD. This correlates with findings from multiple studies investigating decrease in ECD following cataract and glaucoma surgery [3, 6–12]. This is most likely due to the invasive nature of surgery into the anterior chamber, and the possible damage to the endothelium.

Corneal endothelial cells were found to decrease with smoking in this study. Although this is collaborated in a previous study by Sakai et al. in Japan [13], other studies that attempted to evaluate the effect of chronic smoking, did not find an effect [14, 15]. Smoking is also a modifiable factor. People who smoke could be given adequate counselling, so that they can change their lifestyle.

Our results showed that there was a reduction in corneal endothelial cells (132 endothelial cells) in patients that had arcus senilis. However, the level of evidence for this relationship was marginal. This could have been due to the wide confidence interval (-283.6, 19.5), limiting its significance. Arcus senilis is one of the most obvious and easily visualized clinical sign, done with a torch, or slit lamp. It can therefore be a potential indicator of low ECD, however our data was imprecise and more research needs to be done on this.

Most importantly, we found ocular biometric characteristics that were linearly associated with a higher number of corneal endothelial cells, such as corneal diameter, axial length and central corneal thickness. These are part of the routine pre-operative ocular biometry measurements that are readily available in most facilities.

Knowledge of the above factors could be used by clinicians to estimate the corneal ECD of patients especially those in low resource settings where a confocal or specular microscope is not readily available. For example, patients with previous history of ocular surgery, history of smoking, old age, dry eye syndrome, and arcus senilis, have a higher risk of low corneal ECD. Therefore, such patients should be counselled adequately about the visual prognosis, before any intraocular surgery, and also preferably, a more experienced surgeon should operate on such patients.

### Limitations

- Our study was carried out at a big referral eye hospital in Southwestern Uganda, making the study prone to selective referral bias, and therefore findings are not representative of the general population.
- Numbers were not big enough to give a conclusion on the ECD of the normal population, but could be indicative.
- These findings may not necessarily apply to populations of different racial backgrounds, because the study was conducted among individuals of Black African descent.
- To diagnose dry eye, we could have used other tests like punctate staining, and therefore the number of those who had tear film instability, could have been under estimated.
- A contact confocal microscope was used for this study, and therefore some participants declined examination of the second eye, because of the discomfort experienced from direct contact of the cornea, with the confocal cap.
- The results of this study, especially with regards to the correlates of ECD, could be considered preliminary, thus requiring further prospective studies, well knowing the lack of temporal relationship between independent variables and ECD, that is expected in cross sectional study designs.

## Conclusion

This study was conducted in a predominantly black African population, established baseline normal ranges of ECD counts, and found a small proportion of low ECD in our population. Our study was the first of its kind in the region to study corneal endothelial cell density, therefore provided valuable baseline values for informing future research.

Lack of advanced equipment across many centers in Africa notwithstanding, a pragmatic solution would be to develop a clinical tool, which general ophthalmologists could use to estimate the corneal endothelial cell density, using clinical signs that can be easily visualized during routine pre-operative assessment on a slit lamp. This would help to provide correct counseling to patients, before any invasive ocular surgery. We identified important ocular biometric and general lifestyle factors that seemed to influence ECD in this population. Our group plans to develop a predictive model that could utilize these parameters to estimate the corneal ECD at a low cost, compared to the expensive endothelial cell count equipment.

## Data Availability

The data sets used during the current study are available from the corresponding author on reasonable request.

## Abbreviations

AC: Anterior Chamber
ACD: Anterior Chamber Depth
AXL: Axial length
CCT: Central Corneal Thickness
DES: Dry Eye Syndrome
ECD: Endothelial Cell Density
IOP: Intraocular Pressure
K: Corneal curvature
LE: Left Eye
MCD: Mean Cell Density
MURHEC: Mbarara University and Referral Hospital, Eye Centre
RE: Right Eye
OPD: Out Patient Department
PEX: Pseudoexfoliation
